# A mind you can count on: validating breath counting as a behavioral measure of mindfulness

**DOI:** 10.3389/fpsyg.2014.01202

**Published:** 2014-10-24

**Authors:** Daniel B. Levinson, Eli L. Stoll, Sonam D. Kindy, Hillary L. Merry, Richard J. Davidson

**Affiliations:** Waisman Laboratory for Brain Imaging and Behavior, Center for Investigating Healthy Minds, Psychology Department, University of Wisconsin–Madison, Madison, WIUSA

**Keywords:** mindfulness, mind wandering, task-unrelated thought, attention, meta-awareness, meta-cognition, wanting, working memory training

## Abstract

Mindfulness practice of present moment awareness promises many benefits, but has eluded rigorous behavioral measurement. To date, research has relied on self-reported mindfulness or heterogeneous mindfulness trainings to infer skillful mindfulness practice and its effects. In four independent studies with over 400 total participants, we present the first construct validation of a behavioral measure of mindfulness, breath counting. We found it was reliable, correlated with self-reported mindfulness, differentiated long-term meditators from age-matched controls, and was distinct from sustained attention and working memory measures. In addition, we employed breath counting to test the nomological network of mindfulness. As theorized, we found skill in breath counting associated with more meta-awareness, less mind wandering, better mood, and greater non-attachment (i.e., less attentional capture by distractors formerly paired with reward). We also found in a randomized online training study that 4 weeks of breath counting training improved mindfulness and decreased mind wandering relative to working memory training and no training controls. Together, these findings provide the first evidence for breath counting as a behavioral measure of mindfulness.

## INTRODUCTION

James (1890), a founder of American Psychology wrote, “the faculty of voluntarily bringing back a wandering attention over and over again is the very root of judgment, character, and will. … An education which should improve this faculty would be *the* education *par excellence.*” In the 1960s and more recently, others have productively followed James’s interest in wandering attention – under the overlapping terms of mind wandering, task-unrelated-thought (TUT), and stimulus-independent thought – to document that it occurs 30–50% of daily life (Klinger and Cox, 1987; Killingsworth and Gilbert, 2010), and is associated with cognitive task errors (Antrobus, 1968) and worse mood (Killingsworth and Gilbert, 2010; Wilson et al., 2014; but see Franklin et al., 2013).

In contrast, research on the education of voluntarily bringing back a wandering mind has evoked both promise and controversy. Regarding its promise, the practice of returning attention to the present, which is core to mindfulness, has been associated with reduced pain (Zeidan et al., 2011), improved attention (Brefczynski-Lewis et al., 2007), and enhanced well-being (Brown and Ryan, 2003; Tang et al., 2007) among other benefits (Hölzel et al., 2011).

Nonetheless, mindfulness measurements are controversial. For example, self-report on the Five Factor Mindfulness Questionnaire (FFMQ; Baer et al., 2006) cannot distinguish individuals receiving Mindfulness Based Stress Reduction vs. a validated active control intervention (MacCoon et al., 2012) because both interventions increase reported mindfulness equally (MacCoon, personal communication). In addition, mindfulness trainings and monetary incentives equally increase certain cognitive test scores, suggesting that the demand characteristics inherent in mindfulness training studies may result in training studies measuring effects of non-specific factors such as motivation as opposed to, or at least in addition to, mindfulness (Jensen et al., 2012). Therefore, it is unclear the extent to which mindfulness *per se* is captured by self-report or responsible for improvements following putative mindfulness trainings.

It is therefore critical for the field to establish a behavioral and thus less biased measure of mindfulness. Unlike questionnaires, which suffer from retrospective distortions and susceptibility to implicit demand characteristics (e.g., pressure on meditators to report being mindful), behavioral measures prevent “faking good” as ability must be demonstrated instead of simply averred. A behavioral measure could also avoid the confounding, non-specific training effects introduced in mindfulness training studies and provide a more efficient assessment. However, to our knowledge, no behavioral measure of mindfulness exists for scientific use. To address this gap, we present the first validation of such a measure.

### DEFINING AND OPERATIONALIZING MINDFULNESS

We chose *present moment awareness* as a definition of mindfulness to operationalize. Grounded in traditional descriptions of mindfulness (Supplementary Material Introduction), it is a commonality in the diversity of modern scientific definitions (e.g., Brown and Ryan, 2003; Bishop et al., 2004; Baer et al., 2006; Schooler et al., 2011) and meditation styles, which variably emphasize non-attachment, non-judgment, or other facets as well.

Mindfulness of breathing can be indexed by breath counting, which lends itself to objective behavioral study and draws face validity from its longstanding use in mindfulness practice (recorded c. 430 AD, Buddhaghosa, 2010). Prima facie, accurately counting breaths operationalizes mindfulness because it depends on (1) directly perceiving the experience of breathing in the present and (2) awareness that experience (such as mind wandering) is happening, which enables a return of attention to the breath whenever attention drifts. Therefore, although counting is not necessary for mindfulness, we propose mindfulness contributes to accurate breath counting.

### EVALUATING THE CONSTRUCT VALIDITY OF BREATH COUNTING AS AN INDEX OF MINDFULNESS

To test the proposition that breath counting measures mindfulness, we followed the recommendations of Cronbach and Meehl (1955) for establishing such construct validity. We reasoned that if breath counting measures mindfulness, then those skilled in breath counting should exhibit all the theorized consequences of mindfulness, including more meta-awareness, less mind wandering, better mood, and greater non-attachment. The theory behind each of these links in mindfulness’s nomological network is briefly reviewed.

#### Evaluating convergent validity

Mindfulness is not the absence of stimulus-independent thought. Rather, both can coexist according to traditional mindfulness styles with instructions to be aware of the present moment experience of stimulus-independent thoughts arising and passing: “(one skilled in mindfulness) knows a distracted mind to be ‘distracted’... a lustful mind to be ‘lustful’... an angry mind to be ‘angry”’ (Anālayo, 2003). Mindfulness, then, should associate with greater meta-awareness (Fox et al., 2012), particularly of emotions (Nielsen and Kaszniak, 2006) and mind wandering, where meta-awareness is defined as the explicit recognition of the current contents of consciousness (Schooler et al., 2011). Therefore, we assessed the convergent validity of breath counting with meta-awareness in Study 1.

Although mindfulness is the presence of present moment awareness rather than the absence of stimulus-independent thought, in certain contexts mindfulness should result in lessened stimulus-independent thought. For example, when one intends to fully attend to an activity involving minimal discursive thought – e.g., mindfulness of breathing – then awareness of task-unrelated thoughts and their causes (e.g., certain emotions) may lead to their decrease (see Discussion and Schooler et al., 2011). In a similar fashion, mindfulness should likewise attenuate task-unrelated thoughts that purportedly lower mood (>50% of mind wandering, Killingsworth and Gilbert, 2010) if they are understood as unnecessary. In support of these theories, previous research has shown self-reported mindfulness is inversely correlated with mind wandering as indexed by the sustained attention to response task (SART; Allan Cheyne et al., 2009; Mrazek et al., 2012) and positively correlated with well-being (Brown and Ryan, 2003). Therefore, if breath counting accuracy measures mindfulness it should associate with less mind wandering during breath counting and overall, as well as with better mood. We assessed breath counting’s convergent validity with mood in Study 2, and with mind wandering in all studies.

Just as the increased awareness of mindfulness may help lessen stimulus-independent thought, it may also attenuate the influence of certain emotions (Creswell et al., 2007; Brown et al., 2013), such as wanting (Berridge et al., 2009). Indeed, awareness decreases the power of erotica to capture attention (Jiang et al., 2006) and lessens the emotion-induced influences of weather on life satisfaction (Schwarz and Clore, 1983). Therefore, accurate breath counting should associate with non-attachment as demonstrated by a decreased influence of wanting. This prediction is in line with non-attachment’s positive association with mindfulness in traditional theory (Anālayo, 2003) and self-report research (Sahdra et al., 2010). We tested the convergent validity of breath counting with non-attachment in Study 3.

#### Evaluating discriminant validity

In parallel to its convergent validity, we assessed breath counting for discriminant validity by examining its empirical alignment with the theoretical distinctions between mindfulness and established attention constructs such as sustained attention and working memory capacity. Mindfulness practice emphasizes the direct perception of present moment experience, which is a continuously present and changing process (e.g., the felt experience of breathing). In contrast, sustained attention tasks such as the SART emphasize the conceptual detection of infrequent and discrete target content (e.g., detecting a “3” present <5% of total task time). In further contrast, working memory tasks such as the automated operation span task (OSPAN) emphasize the priority-driven maintenance and manipulation of information not present in the current environment (e.g., a string of letters). While each of the three tasks measure an attentional trait by assessing how well a person can maintain a certain attentional set (e.g., holding seven letters in memory while doing math), breath counting should not be highly correlated with the OSPAN or SART, a prediction we assessed in Studies 1 and 2, respectively. Although the SART as an index of mind wandering would ideally be somewhat inversely correlated with breath counting, breath counting’s predicted correlates (e.g., history of meditation practice) should nonetheless remain significant correlates after controlling for the SART, a claim we tested in Studies 2 & 3. Furthermore, although breath counting ability should be stable over time in the absence of intervention (assessed in Study 2), it should be selectively increased by a mindfulness intervention but unchanged by an intervention aimed to increase working memory (assessed in Study 4).

#### Evaluating criterion validity

Following Cronbach and Meehl (1955), we also assessed breath counting’s criterion validity. As they noted, two indices that measure a similar construct should correlate. Therefore, we evaluated whether individuals reporting greater mindfulness on existing mindfulness questionnaires counted breaths more accurately as well (Study 1). We additionally tested for expected group differences by assessing whether long-term meditators counted breaths more accurately than controls (Study 3).

### EVALUATING INCREMENTAL VALIDITY

Finally, we assessed breath counting’s incremental validity relative to extant criteria by testing whether breath counting could explain individual differences in meta-awareness, mind wandering, and non-attachment beyond what could be explained by mindfulness questionnaires (Studies 1 and 3).

## RESULTS

### STUDY 1

In Study 1 we explored the convergent, discriminant, criterion, and incremental validity of breath counting by assessing its correlation with meta-awareness, mind wandering, working memory, and trait mindfulness. We instructed 120 participants to “be aware… of the movement of breath” and count their breaths from 1 to 9 repeatedly. With breaths 1–8 they pressed one button, and on breath nine they pressed another, measuring counting accuracy. Every ∼90 s (60–120 s range) experience sampling probed state mind wandering and meta-awareness, respectively, with 2 6-point Likert scales, “just now where was your attention? (completely on-task/off-task)” and “how aware were you of where your attention was? (completely aware/unaware).” Participants were then probed for their count.

Accurate breath tracking was physiologically confirmed in a subset of 52 participants, with mean keypress rate tracking mean breath rate, *r* = 0.99. In addition, in the total sample, mean keypress rate did not explain counting performance, *r* = -0.04, *P* = 0.67, which showed an average error rate of 22% (SD 15%) with a mean of 29% of errors being self-caught.

**FIGURE 1:**
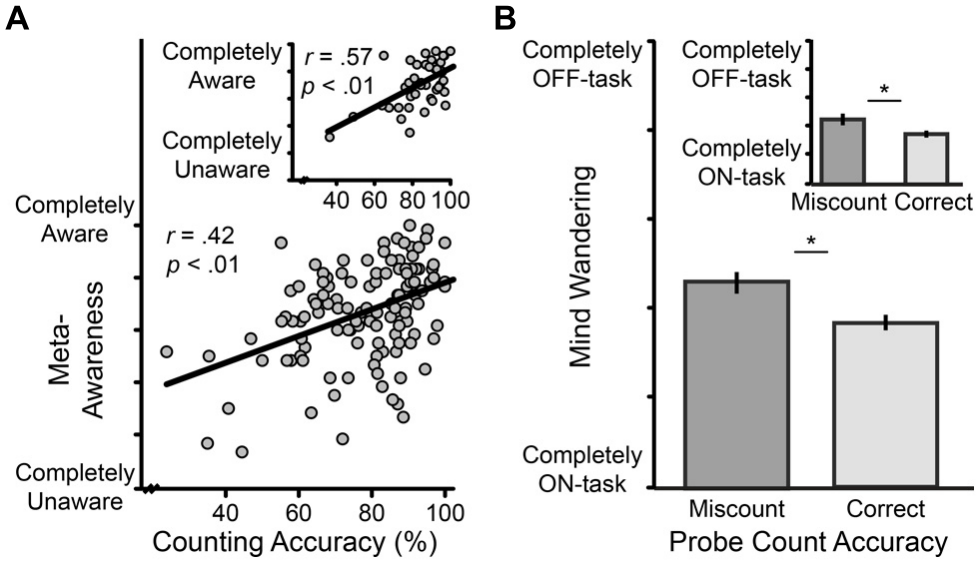
**Cognitive correlates of breath counting accuracy, and their replication (*Insets*). (A)** The relation across participants between state meta-awareness and counting accuracy. State meta-awareness was indexed as the average response to 12 probes during breath counting asking “How aware were you of where your attention was?” on a 6-point Likert scale ranging from “completely aware” to “completely unaware.” Counting accuracy was indexed as the percent of total task count sets correct. **(B)** The relation within participants between momentary mind wandering and counting accuracy. During breath counting participants were randomly probed 12 times for their current count and mind wandering status on a 6-point Likert scale ranging from “completely on-task” to “completely off-task.” For each participant, mind wandering scores were averaged separately for moments when on-count vs. off-count, and then entered into group-level “correct” and “miscount” means displayed by bar graph ( ±1 SE). *p < 0.05.

Guided by theory that those with greater mindfulness experience greater meta-awareness, total task counting accuracy and state meta-awareness during breath counting were correlated across participants. In line with theory, skill in breath counting associated with greater meta-awareness, *r* = 0.42, *P* < 0.001 (**Figure 1A**). Breath counting accuracy also associated with less state mind wandering across participants, *r* = -0.38, *P* < 0.001, as predicted for a valid measure of mindfulness.

To examine these relationships at a finer timescale *within participants*, we investigated whether increased meta-awareness and diminished mind wandering were occurring in the very moments when mindfulness was present. We compared average meta-awareness ratings from correct vs. incorrect count probes within participants and found that moments of accurate counting (vs. miscounting) associated with increased meta-awareness, *t*_(101)_ = 2.51, *P* = 0.01. Mind wandering also decreased during moments of mindfulness indexed by accurate counting, *t*_(101)_ = 4.02, *P* < 0.001 (**Figure 1B**). To confirm findings were not due to probe order, we replicated them in a separate block of 44 participants collected part way through Study 1 who received their count probes preceding TUT probes in an otherwise identical task (Supplementary Material Results and **Figures 1A,B** insets).

When we changed the probe order, we also expanded our experiment battery to end with collecting from participants (*n* = 93) a measure of working memory, the OSPAN (described in Unsworth et al., 2005; Supplementary Material Methods), and two questionnaire measures of trait mindfulness, the Mindful Attention and Awareness Scale (MAAS; Brown and Ryan, 2003; Supplementary Table S1) and the FFMQ (Baer et al., 2006; Supplementary Table S1). Supporting discriminant validity, we found breath counting accuracy uncorrelated with working memory capacity as measured by OSPAN, *r* = 0.04, *P* = 0.71. Supporting criterion validity, we found breath counting accuracy positively correlated with trait mindfulness as reported on the MAAS, *r* = 0.20, *P* = 0.05, and FFMQ, *r* = 0.21, *P* = 0.05 (see Supplementary Table 3 for subscale correlations). Regarding incremental validity, when the MAAS and FFMQ were entered with breath counting into a simultaneous regression for explaining state meta-awareness, counting accuracy still significantly and uniquely explained variance in meta-awareness, *rs* = 0.45, *P* < 0.001. The same was true for mind wandering, *rs* = 0.46, *P* < 0.001.

### STUDY 2

Study 2 investigated breath counting’s convergent validity with mood and overall mind wandering, its discriminant validity relative to sustained attention, and its test–retest reliability, as measures of attentional traits are expected to be stable over time. A new sample of 137 participants completed the state Positive and Negative Affect Scale (PANAS; Watson et al., 1988; seven PANAS scores lost to technical malfunction) followed in counterbalanced order by the go/no-go SART (Robertson et al., 1997; Supplementary Material Methods and Supplementary Figure S1) and a breath counting task. Trait mind wandering scores from the Imaginal Process Inventory (IPI; Singer and Antrobus, 1972; Supplementary Table S1) were available in a pre-existing survey database for 85 of them. Of those participants who performed breath counting as their first task, 54 did an identical breath counting task 1 week later to assess test–retest reliability.

Accurate breath tracking was again physiologically confirmed in a subset of 69 participants, where mean keypress rate tracked mean breath rate, *r*= 0.99. In the total sample, average error rate was 16% (SD 15%) with a mean of 35% of errors being self-caught. Unlike in Study 1, in Study 2 those with slower keypress rates miscounted more, *r* = -0.19, *P* = 0.03. All findings below remain significant when controlling for keypress rate unless otherwise stated.

**FIGURE 2:**
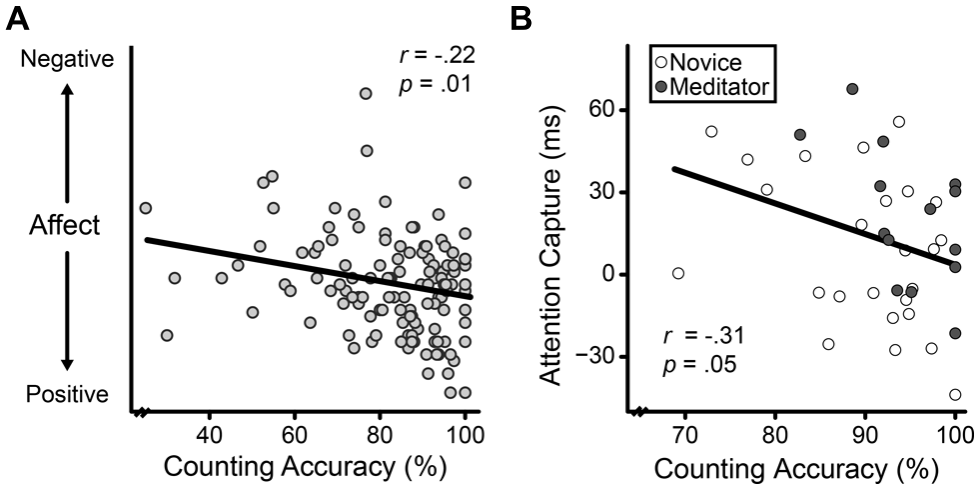
**Affective correlates of breath counting accuracy, indexed as the percent of total task count sets correct. (A)** The relation across participants between state affect (negative–positive) from the Positive And Negative Affect Scale and counting accuracy. **(B)** The relation across all participants between attention capture (defined as response time when reward-associated distractors were present minus response time when they were absent) and counting accuracy.

Supporting breath counting’s convergent validity, counting accuracy associated with better mood, where mood was indexed by a composite of negative minus positive affect, *r* = -0.22, *P* = 0.01 (**Figure 2A**). When positive and negative affect were simultaneously regressed on breath counting accuracy, accuracy independently correlated with both more positive affect, *rs* = 0.17, *P* = 0.05, and less negative affect, *rs* = -0.17, *P* = 0.05. After controlling for keypress rate, however, the correlation with positive affect became non-significant, *rs* = 0.15, *P* = 0.07.

Further substantiating breath counting’s convergent validity, counting accuracy associated with less overall mind wandering. This was true regardless of whether mind wandering was measured with the IPI, *r* = -0.27, *P* = 0.01, or SART indices validated as indirect measures of mind wandering (Allan Cheyne et al., 2009), namely errors of commission, *r* = -0.19, *P* = 0.03, and RT variability, *r* = -0.32, *P* < 0.001. Importantly, SART indices were far from perfectly correlated with breath counting, supporting its discriminant validity. Moreover, counting accuracy’s relationship with overall mood and with mind wandering as indexed by the IPI remained significant after controlling for individual differences in SART errors of commission – *rs*= -0.25, *P* = 0.005 and *rs*= -0.24, *P* = 0.03, respectively – suggesting that breath counting’s relationship with mood and mind wandering is not simply a result of individual differences in sustained attention.

**FIGURE 3:**
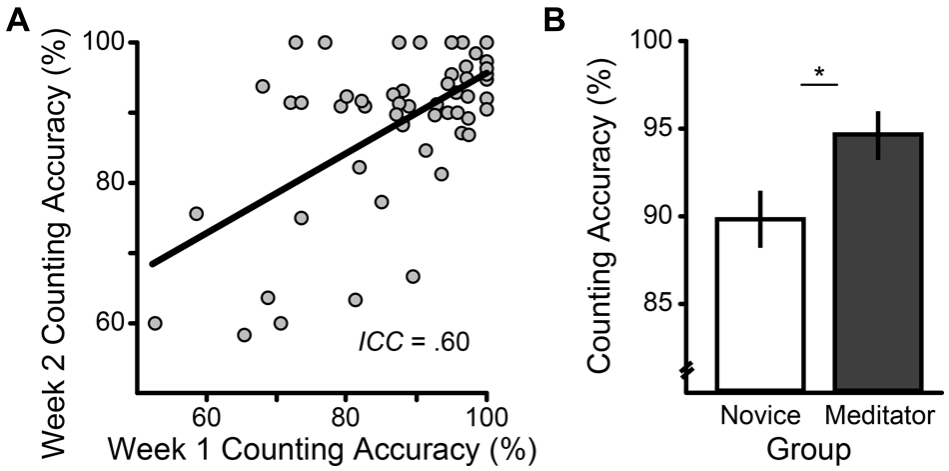
**Reliability and known-groups validity of breath counting accuracy, indexed as the percent of total task count sets correct. (A)** 1 week test–retest reliability. **(B)** Long-term vs. age-matched novice meditators’ counting accuracy (±1 SE); the group difference remained significant after controlling for individual differences in sustained attention indexed by sustained attention to response task (SART) commission errors. *p < 0.05.

Breath counting demonstrated a 1-week test–retest reliability of ICC = 0.60 (**Figure 3A** and Supplementary Material Results).

### STUDY 3

Mindfulness is thought to associate with non-attachment, typified in part by a decreased influence of wanting. Wanting, defined as an incentive motivation to approach, can be irrationally incongruent with cognitive goals, e.g., as occurs in addiction (Berridge et al., 2009). When approach contradicts cognitive goals and is unhelpful, it indexes wanting with particular clarity. Therefore, to measure the influence of wanting we assessed how much individuals were slowed by attending to a distractor formerly paired with reward despite their cognitive goal of completing a visual search as quickly as possible. This validated measure of attention capture (Anderson et al., 2011) parallels the paradigm of operant extinction used to measure wanting in animals (e.g., Wyvell and Berridge, 2000), and so we predicted it would correlate inversely with breath counting accuracy – supporting convergent validity – and do so beyond what could be explained by self-reported mindfulness – supporting incremental validity.

As described in detail elsewhere (Anderson et al., 2011; Supplementary Material Methods and Supplementary Figure S2), for the training portion of the attention capture task participants were monetarily rewarded when they accurately identified targets highlighted by specific colors. Later, during the testing portion, participants were told to ignore color as irrelevant and no rewards were given. Targets were instead highlighted by distinct shapes among distractors, and participants identified targets by keypress “as quickly as possible while minimizing errors.” On half of the trials one distractor was a color previously associated with reward. Attention capture scores were calculated by subtracting the average RT in trials with reward-associated color distraction from the average RT in trials without such distraction (as in Anderson et al., 2013b).

Replicating previous research, the presence of a formerly rewarded distractor successfully captured attention as demonstrated by significantly slower RTs on trials with stimuli previously paired with reward (vs. not), *t*_(38)_ = 2.99, *P* < 0.01. To assess breath counting’s convergent validity with non-attachment as exemplified by a decreased influence of wanting, we correlated counting accuracy with individual differences in the extent of attentional capture. We found that greater accuracy was associated with less capture, *r* = -0.31, *P* = 0.05 (**Figure 2B**), suggesting that breath counting ability is related to a reduced influence of wanting, as expected for a measure of mindfulness. In addition, in support of its incremental validity, breath counting accuracy remained a significant predictor of attention capture when entered in a simultaneous regression with the FFMQ, *rs* = 0.38, *P* = 0.02.

The participants we recruited for Study 3 were both long-term and novice meditators, as this population allowed us to simultaneously address a second aim of evaluating expected group differences in breath counting. We also took the opportunity to more deeply probe the discriminant validity of breath counting by evaluating whether its predicted covariation with meditation history could be explained merely by individual differences in sustained attention. We found that long-term meditators, purportedly skilled in mindfulness, displayed greater counting accuracy, *t*_(36)_ = 2.23, *P* = 0.03 (**Figure 3B**), and less mind wandering, *t*_(36)_ = 2.11, *P* = 0.04, than age-matched novice meditators. Importantly, the group difference in counting accuracy remained significant after controlling for SART commission errors, *t*_(35)_ = 2.01, *P* = 0.05, suggesting that breath counting measures skill in mindfulness beyond that accounted for by sustained attention.

### STUDY 4

Study 4 further tested breath counting for discriminant validity by assessing its selective sensitivity to mindfulness training interventions. We reasoned that if breath counting measures mindfulness, than an individual’s counting accuracy should increase following training in mindfulness but not training in working memory, a construct found uncorrelated with breath counting accuracy in Study 1.

We drew training methodology from a growing literature suggesting that mental capacities such as working memory can be improved with practice, as evidenced by neural plasticity and better performance on working memory measures following repeated practice of working memory tasks such as the spatial n-back task (Jaeggi et al., 2008; McNab et al., 2009; Davidson and McEwen, 2012; but see Redick et al., 2013). In the same way that working memory may be improved by repeated practice of working memory tasks, mindfulness may be improved by repeated practice of breath counting if it is indeed a mindfulness task. Therefore, in a randomized controlled trial we tested whether breath counting training – but not n-back training or no training – could increase counting accuracy and self-reported mindfulness as well as decrease mind wandering.

**FIGURE 4:**
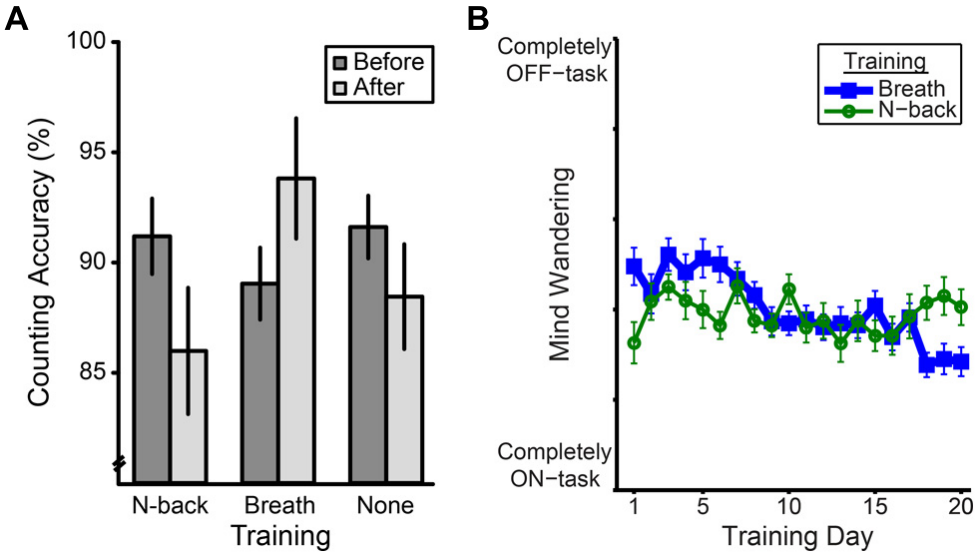
**Breath counting as a method and measure of mindfulness training. (A)** Counting accuracy for each training group before and after the training period (± 1 SE), indexed as the percent of total task count sets correct. **(B)** Change in mind wandering over the course of 20 consecutive weekdays of either breath counting or n-back training. Mind wandering during daily training was calculated from the average of two 25 min training sessions (AM and PM). Mind wandering was measured by a single rating at the end of each session that answered the question “Where was your attention just now?” on a 6-point likert scale ranging from “completely on-task” to “completely off-task.” Error bars represent within participants’ ± 1 SE.

Participants were randomized into three training groups, a breath counting training, a spatial n-back training control, and a no-training control. Attrition rates were 27%, 33%, and 15%, making final sample sizes of 22, 20, and 29, respectively (see Supplementary Material Methods and Supplementary Figures S3 and S4 for training protocols and retention details at each study phase; online breath counting training can be viewed at http://webtasks.keck.waisman.wisc.edu/b/demo). For 4 weeks, breath counting and n-back trainees completed two 25 min trainings each weekday which ended with a mind wandering thought probe “just now where was your attention? (completely on-task/off-task).” When comparing the last 2 weeks to the first 2 weeks of training, both active training groups improved in training performance (Supplementary Figure S6), but only breath counting participants decreased in mind wandering, group × time interaction *F*_(2,40)_ = 7.02, *P* = 0.01, simple main effect of time for breath counting participants *F*_(1,40)_ = 25.18, *P* < 0.001, simple main effect of time for n-back participants *F*_(1,40)_ = 1.26, *P* = 0.27 (**Figure 4A** and Supplementary Material Methods), as expected for a mindfulness training. While the groups did not at first significantly differ in mind wandering (simple main effect of group during first 2 weeks, *F*_(1,40)_ = 1.46, *P* = 0.24), the initial numerically lower mind wandering during n-back performance was unsurprising given that, independent of training, such demanding working memory tasks suppress mind wandering (McKiernan et al., 2006).

Before and after the 4-week training period, all three groups completed testing including the FFMQ, a breath counting task with mind wandering probes, and a verbal 3-back task. In line with the hypothesis that repeated breath counting trains mindfulness, we found a group × time interaction in FFMQ scores, *F*_(2,68)_ = 4.83, *P* = 0.01, such that although the two control groups did not significantly differ from each other in their pre–post change in trait mindfulness, *F*_(1,68)_ = 0.02, *P* = 0.88, the breath counting group increased in trait mindfulness relative to the two control groups, *F*_(1,68)_ = 9.63, *P* < 0.01.

As evidence that breath counting accuracy measures mindfulness, we also found a group × time interaction trend in counting accuracy, *F*_(2,68)_ = 2.97, *P* = 0.06, and an interaction in mind wandering, *F*_(2,68)_ = 5.09, *P* < 0.01, during the breath counting task. Specifically, planned comparisons revealed that the two control groups did not significantly differ from each other in their pre–post change in counting accuracy, *F*_(1,68)_ = 0.24, *P* = 0.63 (**Figure 4B**), or mind wandering, *F*_(1,68)_ = 3.75, *P* = 0.06. However, the breath counting group demonstrated decreased mind wandering, *F*_(1,68)_ = 5.40, *P* = 0.02, and improved counting accuracy, *F*_(1,68)_ = 5.89, *P* = 0.02, relative to the two control groups (**Figure 4B**). Moreover, within the breath counting group, those who increased most in counting accuracy as a result of training were also the ones who increased most in FFMQ trait mindfulness, *r* = 0.44, *P* = 0.04.

## DISCUSSION

While self-report measures have provided a helpful beginning for assessing mindfulness, to date a behavioral measure immune to biases inherent in self-report is still lacking. Here we validate breath counting as a behavioral measure of mindfulness with findings that extend previous research (Lutz et al., 2009; Sahdra et al., 2010; Braboszcz and Delorme, 2011; Burg and Michalak, 2011; Hasenkamp et al., 2012; Brown et al., 2013; Mrazek et al., 2013; Baird et al., 2014). We found that breath counting accuracy tracked with naturally occurring variations in self-reported mindfulness, distinguished well-practiced meditators from novices, and increased following a mindfulness training.

### CONVERGENT VALIDITY: EVIDENCING BOTH THEORY AND METHODS

We also provided the first evidence that skill in mindfulness rigorously measured through behavior is related to more meta-awareness, less mind wandering, better mood, and greater non-attachment, in line with theoretical claims that underlie explanations of mindfulness’s educational and health benefits. Our novel assessment of mindfulness’s relation to non-attachment using attention capture especially highlights non-attachment as a mechanism by which mindfulness may ease addiction (Tang et al., 2013; Bowen et al., 2014), a disorder in which reward-associated attention capture is elevated (Anderson et al., 2013a). Such convergence of breath counting and mindfulness theory helps substantiate both per Cronbach and Meehl (1955): “we do not first ‘prove’ the theory, and then validate the test, nor conversely.... Actually the evidence is significant for all parts.”

One hypothesis to be explored for the convergence of mindfulness with these constructs is awareness-dependent learning and memory. As a specific example, present moment awareness of task-unrelated worry and its consequences – such as forecasting dangers that do not occur (Gilbert and Wilson, 2009) – may form a memory that the worry was unnecessary. In the future, meta-awareness of worry may retrieve that memory, reducing the priority of worrying and thus the working memory resources maintaining it (Levinson et al., 2012). Reducing such mind wandering would reduce the negative emotions it triggers (Killingsworth and Gilbert, 2010), improving mood. And with fewer negative emotions to fuel it (Horowitz and Becker, 1973), mind wandering would reduce further. The same would apply to wanting, resulting in non-attachment.

### DISCRIMINANT VALIDITY: CLARIFYING THE CONSTRUCT OF MINDFULNESS

To establish the validity of a new construct, it must be distinguished from existing constructs. Our data suggest that mindfulness as indexed by breath counting is not reducible to mind wandering’s absence, working memory, or sustained attention, as is evident from the variance in breath counting accuracy unexplained by these measures. How then is mindfulness unique?

Mindfulness encourages awareness that task-unrelated thoughts are happening as present moment experiences. As a result, mindfulness, and task-unrelated thoughts may coexist. At the same time, since mindfulness encourages direct perception of present experience, we suggest mindfulness may simultaneously reduce task-unrelated thought as a natural byproduct of more fully saturating perceptual resources (Supplementary Material Discussion; Forster and Lavie, 2009; Levinson et al., 2012). This perspective can account for the inverse relation we found between counting accuracy and mind wandering without defining mindfulness by the absence of task-unrelated thought.

Such reduction of mind wandering is putatively independent of working memory (Lavie et al., 2004; Forster and Lavie, 2009), further distinguishing mindfulness from working memory tasks and the SART which depend on working memory to block task-unrelated thoughts from awareness (McVay and Kane, 2009). Therefore, this perspective can also explain why mindfulness and working memory capacity are uncorrelated (Supplementary Material Discussion), why n-back training did not improve breath counting accuracy, and why breath counting significantly differentiated long-term meditators and novices even after controlling for individual differences in sustained attention indexed by SART errors. Mindfulness also differs from sustained attention in that it theoretically changes one’s relationship with emotions, in line with findings that breath counting accuracy (but not SART errors, *r* = 0.11, *P* = 0.51) predicted less reward-associated distraction.

### CRITERION AND INCREMENTAL VALIDITY

Cronbach and Meehl (1955) observed that construct validity evolves by bootstrapping, wherein a new test is initially validated with existing imperfect tests (e.g., self-report) yet may be ultimately judged to have greater construct validity. For example, the thermometer received initial validation from self-reports of felt temperature, but ultimately outperformed self-reported temperature in predicting the pressure of a heated gas. We too validated breath counting using existing methods – mindfulness training and self-report – and so it is important to discuss how we navigated their limitations and how breath counting compares with them in predicting the theoretical correlates of mindfulness.

In theory, training effects result from increasing a targeted quality (e.g., mindfulness). Yet in practice training effects can result from untargeted, non-specific factors such as trainees’ group interactions or motivation, as has been previously found in mindfulness trainings (e.g., Jensen et al., 2012). Our methods minimized such factors (Supplementary Material Discussion). Evidencing this, we found verbal 3-back performance improved most following n-back training [group × time interaction, *F*_(2,68)_ = 3.72, *P* = 0.03; improvement in n-back training group vs. breath counting and no-training groups, *F*_(1,68)_ = 6.86, *P* = 0.01] but did not differ following breath counting vs. no training (*F*_(1,68)_ = 0.32, *P* = 0.58). This suggests the effects of our mindfulness training were not simply due to non-specific factors such as motivation that should have improved performance non-selectively on any task, including the verbal 3-back.

As mentioned, self-report is vulnerable to confounds such as retrospective bias and demand characteristics. We protected against spurious correlations between self-report and breath counting via replication with bias-resistant methods, as illustrated in our mind wandering data. For example, to decrease retrospective bias, participants reported on mind wandering occurring in the moment instead of the past using experience sampling methods that demonstrate convergent validity with neural measures (Smallwood et al., 2008; Christoff et al., 2009). To decrease demand characteristics, we collected mind wandering reports with the IPI weeks before participants realized they might be in a breath counting experiment and experience any demands. Finally, to sidestep self-report biases altogether, we administered the SART which has been validated as an indirect measure of mind wandering (Smallwood et al., 2008). In all cases, even after self-report bias was reduced, the association between breath counting and mind wandering replicated.

As construct validity progresses, one expects newer measures to display variance that can better predict the theoretical correlates of the construct (Supplementary Material Discussion). In line with this view, using multiple regression we found that breath counting, over and beyond the FFMQ, predicted non-attachment as indexed by decreased attentional capture by reward-associated distractions. Breath counting also significantly explained an individual’s meta-awareness and mind wandering beyond what was possible with the MAAS and FFMQ alone. These data demonstrate breath counting’s incremental validity over existing measures for inferring skill in mindfulness.

### FUTURE DIRECTIONS

Incremental validity notwithstanding, breath counting requires further research on its sensitivity to the more affective dimensions of mindfulness and its specificity to mindfulness vs. established attention constructs. Mindfulness is putatively associated with non-attachment (as evidenced in Study 3) and non-judgment. Therefore, it would be helpful to test, for example, whether decreasing craving increases counting accuracy. Conversely, the validity of breath counting should be questioned if increasing self-judgment about counting errors leads to sustainable increases in counting accuracy. Since breath counting is likely not process pure, it will also be important for future investigations to discriminate what variance in counting accuracy is unrelated to mindfulness and its affective correlates in order to avoid over-interpreting counting accuracy as solely reflecting mindfulness as opposed to other attention constructs as well.

Nonetheless, the present validation of breath counting is a first step in behaviorally measuring mindfulness that opens many avenues for research. As exemplified here, breath counting can now behaviorally evaluate trainings for their impact on mindfulness *per se* and identify which individual differences accompany mindfulness. It can also start a behavioral investigation on the extent to which mindfulness is a domain-general capacity. To take working memory research as an example, the development of behavioral measures with verbal vs. spatial content has clarified that working memory of words vs. spatial location is similar but distinct. While the difference suggests working memory may partly rely on content-specific abilities, the similarity points to a domain-general working memory capacity used to complete both types of tests (Kane et al., 2004). In the same way, breath counting may depend on both breath-specific factors and domain-general mindfulness. Future research correlating breath counting with behavioral measures of mindfulness of diverse content, including emotion, should elucidate the domain-generality and content-specificity of the structure of mindfulness.

Our initial findings suggest breath counting may be useful not only scientifically as a measurement tool but also clinically as a mindfulness training. As a training that simultaneously measures change in skill, it allows evidence-based tailoring of training on an individual basis. In theory, it could determine the guidance that most improves skill for an individual and insert it in the very moment his or her mindfulness lapses. Since the counting errors signaling these lapses occur with greater frequency than trainees notice on their own, such feedback may increase opportunities to practice voluntarily bringing back a wandering attention, a skill William James recognized as fundamental.

### CONCLUSION

For over 1500 years mindfulness trainees have used breath counting for training in mindfulness. Its present adaptation for scientific purposes now enables a rigorous behavioral investigation of the promise mindfulness shows in education (Diamond and Lee, 2011), physical health (Barrett et al., 2012), and well-being (Brown and Ryan, 2003).

## MATERIALS AND METHODS

### STUDY 1

Usable data were collected from 164 participants (62% male; age: mean 22.5, ranging 17–65; 19 excluded: see Supplementary Material Methods for details) from the University of Wisconsin–Madison community paid at $10/hr. Participants gave informed consent and the University of Wisconsin–Madison Institutional Review Board approved procedures. Following a 6 min resting baseline, participants counted breaths from 1 to 9 repeatedly for 18 min. With breaths 1–8 they pressed one button, and on breath nine they pressed another. If they lost count, participants were instructed to press a button reserved for indicating self-caught miscounting and begin again at one with the next breath. Every ∼90 s (60–120 s range) experience sampling probed state mind wandering and meta-awareness, respectively, with 2 6-point Likert scales, “just now where was your attention? (completely on-task/off-task)” and “how aware were you of where your attention was? (completely aware/unaware).” Participants were then probed for their count.

Experience sampling during breath counting yielded a set of 12 TUT ratings and a set of 12 meta-awareness ratings. Each set was averaged to index state mind wandering and state meta-awareness, respectively. For analyses of ratings accompanying correct vs. incorrect count probes, participants without data in both categories (e.g., never off count at probe) were excluded (*n* = 18). Counting accuracy was calculated as the number of correct count sets divided by the total number of count sets, i.e., 100% – (# of incorrect ongoing 9-counts + # of incorrect count probe responses + # of self-caught miscounts) / (# of ongoing 9-counts + # of count probe responses + # of self-caught miscounts).

Throughout Study 1, during breath counting a subset of participants (*n* = 52) wore a respiration belt (Model MP150CE, BIOPAC, Goleta, CA, USA). Mean breath rate was computed as the average time between inhale peaks in the respiration signal.

### STUDY 2

For course credit, a new set of 137 participants with usable data (38% male; age: mean 18.8, ranging 18–26; 11 excluded: see Supplementary Material Methods for details) completed the PANAS and, in counterbalanced order, the SART (Supplementary Material Methods) as well as a 15 min breath counting task without experience sampling. Counting accuracy was calculated as 100% – (# of incorrect ongoing 9-counts + # of self-caught miscounts)/(# of ongoing 9-counts + # of self-caught miscounts). A subset of participants (*n* = 69) wore a respiration belt. Of those participants who performed breath counting as their first task, 54 with usable data (two excluded) returned to lab 1 week later to breath count again. Additionally, we measured 85 participants’ trait mind wandering by including the IPI in a larger mass survey they completed weeks before deciding to enroll in Study 2 (see Supplementary Material Methods for an example item from the IPI and other questionnaires in Studies 1–4).

### STUDY 3

We recruited a group of 14 long-term meditators (57% male, age: mean 53.6, ranging 29–67) from local Buddhist meditation groups and matched them in age to a group of 25 novice meditators (36% male, age: mean 53.7, ranging 29–68). For the purpose of the present study, a long-term meditator was defined as having practiced meditation formally for at least 30 min a day, 5 days a week for the past 3 years, and possessing a total of 750 + lifetime practice hours. Total practice hours in long-term meditators ranged 850–16700 (median 4288).

Participants were paid $10/h plus in-task earnings to complete an attention capture training (Supplementary Material Methods), the FFMQ, and the SART (one novice SART lost to experimenter error). Participants then returned 3 weeks later for a final visit in which they completed a refresher attention capture task training followed by a breath counting task similar to that described in Study 1, save that it lasted 30 min, had 10 experience samplings each separated by ∼3 min (1–5 min range), and did not include meta-awareness probes. Finally, participants performed an attention capture testing (Supplementary Material Methods).

### STUDY 4

Of the 113 participants recruited by offering $300 for completing an “attention training study,” 94 completed a pre-test battery (Supplementary Material Methods), including a verbal 3-back task, an 18 min breath counting task without meta-awareness probes or self-caught miscounting [counting accuracy calculated as 100% – (# of incorrect ongoing 9-counts + # of incorrect count probe responses)/(# of ongoing 9-counts + # of count probe responses)], and an FFMQ modified to query experience “in the last 2 weeks” so that the measure would be sensitive to changes that occurred during the 4 weeks of training. Participants were then randomized to breath counting, spatial n-back, or no training (Supplementary Material Methods; http://webtasks.keck.waisman.wisc.edu/b/demo) and returned 4 weeks later to complete an identical post-test battery.

Outcomes of interest administered pre- and post-training were analyzed using an ANOVA with training group (breath counting vs. n-back vs. no training) as a between participant factor and time (Pre vs. Post training) as a within participant factor. Planned contrasts of active training group vs. controls (active control and no-training) on Pre vs. Post training scores were used to follow up on group × time interactions. Mind wandering during trainings sessions was analyzed using an ANOVA with active training group (breath counting vs. n-back) as a between participant factor and time (first half vs. second half of training) as a within participant factor. Training performance was analyzed within each active training group using paired *t*-tests (first half vs. second half of training).

## AUTHOR CONTRIBUTIONS

Under the supervision of Richard J. Davidson, Daniel B. Levinson designed, analyzed, and wrote up the research. Eli L. Stoll, Sonam D. Kindy, and Hillary L. Merry collected data and assisted in writing and analysis.

## Conflict of Interest Statement

The authors declare that the research was conducted in the absence of any commercial or financial relationships that could be construed as a potential conflict of interest.
