# Supplementary material for: A mind you can count on: validating breath counting as a behavioral measure of mindfulness
Source: Front Psychol. 2014 Oct 24;5:1202. doi: 10.3389/fpsyg.2014.01202 (PMC4208398; doi:10.3389/fpsyg.2014.01202)
Supplement: Supplementary file 1 [file Data_Sheet_1.DOCX]

***Supplementary Material***

**A mind you can count on: validating breath counting as a behavioral measure of mindfulness**

**D.B. Levinson^1^*, E.L. Stoll^1^, S.D. Kindy^1^, H.L. Merry^1^, R.J. Davidson^1^**

^1^Waisman Laboratory for Brain Imaging and Behavior, University of Wisconsin-Madison, Psychology Department, Madison, WI, USA

*** Correspondence:** D.B. Levinson & R.J. Davidson, Waisman Laboratory for Brain Imaging and Behavior, University of Wisconsin-Madison, Psychology Department, 1500 Highland Ave., Madison, WI 53705, USA.

[danlevinson@alumni.stanford.edu](mailto:danlevinson@alumni.stanford.edu)

[rjdavids@wisc.edu](mailto:rjdavids@wisc.edu)

1. **Introduction**

## Defining and operationalizing mindfulness

Present moment awareness encompasses 1) nondistraction from the present (e.g. not being lost in mind wandering) and 2) awareness that experience is happening in the present (including awareness of the experience of mind wandering). Using present moment awareness as a definition of mindfulness has basis in traditional descriptions of mindfulness. For example, commentary on the *Abhidhammattha Sangaha* from the 12^th^ century defines mindfulness or *sati* as “attentiveness to the present” that 1) is characterized by “not floating away from the object” and 2) is manifest as “confronting an objective field” (of present moment experience) in “guardianship” (e.g. from becoming lost in mind wandering) (Bodhi, 1993).

With this definition in mind, we operationalized mindfulness practice in the context of “mindfulness of breathing”, a meditation style requiring present moment awareness.  Its main instruction mirrors the two aforementioned components of mindfulness: 1) to be aware of the experience of breathing (in the present, as opposed to lost in thought) and 2) to be aware when attention has wandered in order to then return attention to the breath. Having said this, we acknowledge that mindfulness is not specific to a particular experience (e.g. the breath) and that mindfulness practice may importantly include facets (e.g. nonjudgment, nonattachment) that may not be as emphasized in mindfulness of breathing as in other mindfulness styles.

1. **Methods**

## Study 1

## Participants

Of the 19 excluded participants, 11 misunderstood instructions, 5 fell asleep, and there were 3 technical malfunctions.

## Operation span task

A full description of the Operation Span can be found elsewhere (Unsworth et al., 2005). In brief, the task consisted of 15 trials. For each trial, the display switched 3 to 7 times between letters for memorization and math equations (e.g. 1 + (3/3) = ?) for verification under response deadline. Each participant’s latencies for 15 math-only practice questions were used to create individualized response deadlines (*M* + 2.5 *SD*s). A participant’s score on the Operation Span was calculated as the sum of the letters recalled in accurate sequence from all 15 trials.

## Breath counting probe order analyses

Experience sampling during breath counting yielded a set of 12 task-unrelated thought (TUT) probes. For probe reaction time (RT) analyses, each of the 12 probes were binned as “on-task” (TUT Likert rating of 4-6) or “off-task” (TUT Likert rating of 1-3), and participants without data in both bins (e.g. never “off-task”) were excluded from probe order analyses (*n* = 29).

## Exploratory measures

The Cognitive Failures Questionnaire was also administered as an exploratory measure (results listed in **Supplementary Table 2**).

## Study 2

## Participants

Of the 11 excluded participants, 5 misunderstood instructions and 6 fell asleep. Of those participants who performed breath counting as their first task, 2 were excluded for misunderstanding instructions.

## Sustained Attention to Response Task

The Sustained Attention to Response Task (SART; Robertson et al., 1997) is a go/no-go task with single digits (1-9) presented every 1150 ms (digit presented for 250 ms followed by a 900 ms mask of an O with an X through it; **Supplementary Figure 1**). Performance was quantified by errors of commission during the 25 no-go trials and by the RT coefficient of variability (RT CV, defined as *SD* of RT / mean RT) during the 200 go trials. Errors of commission are theorized to index gross mind wandering, and RT CV is theorized to be sensitive to mind wandering of the type that occurs in parallel with successful but automated task performance (Allan Cheyne et al., 2009).

## Exploratory measures

The Anger Rumination Scale and Adult ADHD Self Report Scale were also administered as exploratory measures (results listed in **Supplementary Table 2**), with 9 and 11 scores lost to technical malfunction, respectively.

## Study 3

## Attention Capture Task

For attention capture task training and testing (described in detail by (described in detail by Anderson et al., 2011; **Supplementary Figure 2**), participants completed trials in which a 400-600 ms fixation was followed by a ring of 6 different colored shapes and then a feedback display. During training, the shapes were all circles, and the target shape was either red or green. Participants indicated by keypress “as quickly as possible while minimizing errors” and with a 1000 ms response deadline whether the target contained a vertical or horizontal line; nontargets contained diagonal lines. Feedback displayed rewards earned for accurately identifying target line orientation on that trial as well as total rewards earned so far. During testing, participants were informed that color was now irrelevant to the task and should be ignored. Targets were now either a diamond among 5 distractor circles or a circle among 5 distractor diamonds, and participants indicated by keypress within 1500 ms whether targets contained a vertical or horizontal line. On 25% of the trials, one of the distractors was red, on 25% one was green, and on 50% no previously rewarded color – neither red nor green – was present. No rewards were given at testing; feedback only communicated accuracy from the trial just completed. During training and testing, red and green never appeared on the same trial.

Training consisted of 240 trials at visit 1 and 60 trials at the beginning of visit 2. Participants subsequently received 240 trials of testing at the end of visit 2. The locations and colors of targets and distractors were crossed and counterbalanced. Half of participants were assigned red for their high-reward target, half were assigned green. During training, accurate responses to high-reward targets were rewarded with high-reward feedback (10 cents) on 80% of trials and low-reward feedback (2 cents) on 20% of trials. The reverse was true for low-reward targets. As in past research (Anderson, Laurent, & Yantis, 2013), data were collapsed across high and low reward, and an attention capture score was calculated by subtracting the average RT in testing trials on which a red or green distractor was present from the average RT in testing trials without a red or green distractor. RTs more than 2.5 *SD*s above and below the mean RT for each participant were excluded from calculations.

## Exploratory measures

The Barratt Impulsivity Scale was also administered as an exploratory measure (results listed in **Supplementary Table 2**).

## Study 4

## Participants

The study recruited 113 participants with access to internet from the University of Wisconsin-Madison and surrounding community. Email and online advertisements described the duration and compensation ($300) for completing an “attention training study.” Of those who responded to the ads, 94 participants completed pre-testing and were then randomized using a random-number generator into 1 of 3 training groups. A breath counting training was targeted to increase mindfulness with 30 participants. An n–back training was targeted to increase working memory but not mindfulness with 30 participants. For a non-active control, a no-training group simply completed the test battery that all groups completed before and after the 4-week training period with 34 participants. Following attrition, final sample sizes were 22, 20, and 29 for the breath counting training, n-back training, and no-training groups, respectively (**Supplementary Figure 3**). These sample sizes and attrition rates are typical for attention training studies (e.g. Harrison et al., 2013).

## Protocol

On arriving for their first visit, participants completed a testing session including a breath counting task and a verbal 3-back task. Additionally, participants completed questionnaires online from home, including the Five Factors of Mindfulness Questionnaire in which participants were asked to rate themselves based on their experience in the past 2 weeks so that the measure would be sensitive to changes that occurred during the 4 weeks of training.

Participants who completed pre-testing were randomized to the 3 training conditions. Both active training groups trained from their personal computers online for 4 weeks, 5 days/week, for 25 minutes once in the morning and once in the evening each weekday. Two research assistants enrolled participants, randomized them to groups, and tracked their home practice. The assistants discontinued participation of anyone failing to satisfactorily complete at least 75% of training sessions (5 breath counting trainees, 8 n-back trainees). There were no significant deviations from the trial protocol.

After 4 weeks, all groups returned to lab for a post-testing with identical measures to pre-testing. Participants were not told the research hypotheses. Researchers collecting post-test data were not masked to intervention assignment.

## Breath counting training

Participants counted their breaths from one to nine, again and again, for ~25 minutes. With breaths 1-8 they pressed the down arrow, and on breath 9 they pressed the right arrow to end the count set so that counting accuracy could be assessed for each set. Guided mindfulness audio instruction began each session, and feedback was delivered at the end of each count set according to participants’ counting accuracy (e.g. see <http://webtasks.keck.waisman.wisc.edu/b/demo> and <https://webtasks.keck.waisman.wisc.edu/b/8minSilent> for abbreviated versions of the first and last training sessions, respectively). Sessions ended with participants answering the question “Where was your attention just now?” on a 6-point Likert scale ranging from “completely on-task” to “completely off-task”.

## N-back training

Participants performed a spatial adaptive single n-back task for ~25 minutes modified from Jaeggi et al. (2008). Just as dual n-back training, single n-back training has been found effective in improving scores on cognitive tasks (Jaeggi et al., 2014). In n-back training a square appeared every three seconds at a location on the screen. Participants pressed “A” if the square appeared at the same location it appeared n (e.g., 3) appearances before, or “L” if the square appeared at a different location (**Supplementary Figure 4**). At the end of ~75 sec, participants received feedback on their accuracy. They advanced to n+1-back training if accuracy was above 90%, or regressed to n-1-back training if accuracy was below 75%. Sessions ended with participants answering the question “Just now where was your attention?” on a 6-point Likert scale ranging from “completely on-task” to “completely off-task”.

## Breath Counting Task

As in Study 1, but without meta-awareness probes or self-caught miscounting.

## Verbal 3-back

A single letter appeared every three seconds, and participants pressed “A” if the letter was the same as the letter that appeared 3 letters before, or “L” if the letter was different (**Supplementary Figure 5**). Each task block lasted 75 seconds and blocks continued for 15 minutes without feedback.

## Exploratory measures

Exploratory measures were also administered in the pre/post test battery, including the Operation Span task, Backward Digit Span task, Reading the Mind in the Eyes Task, Imaginal Process Inventory (Daydreaming subscale), Beck Depression Inventory, Psychological Wellbeing Scale, Positive and Negative Affect Scale, Spielberger Trait Anxiety Inventory, Acceptance and Action Questionnaire, Penn State Worry Questionnaire, Life Satisfaction Scale, the Self-Compassion Scale, and daily life experience sampling by text messages 8 times per day for 7 days asking “How do you feel?” on a scale of (1)=bad through (9)=good and “Are you thinking of something other than what you are doing?” in a yes/no format (results listed in **Supplementary Table 2**).

1. **Results**

## Study 1

Participants Inspection of probe RTs from the original probe order of Study 1 revealed that participants took longer to report off-task vs. on-task attention during TUT ratings, *t*_(90)_ = 2.43, *P* = .02. Since participants reported their count only after finishing the TUT rating, they needed to remember their count longer when reporting off-task attention.  As a result, it could be argued that breath counting’s association with mind wandering was simply a result of forgetting accurate counts due to taking longer to report mind wandering.

To further clarify breath counting’s association with mind wandering and attempt replication, a separate block of 44 participants collected part way through Study 1 received the count probe first before TUT probes in an otherwise identical task.  Findings replicated, with greater meta-awareness and less mind wandering in those skilled in breath counting, *r* = .57, *P* < .001 (**Figure 1A** inset) and r = -.62, *P* < .001, respectively. Greater meta-awareness and less mind wandering were also found during moments of accurate counting within participants’ performance, *t*_(28)_ = 2.93, *P* < .01 and *t*_(28)_ = 2.06, *P* = .05 (**Figure 1B** inset), respectively (for these within-participant analyses, participants who were either never off count or never on count at probe were excluded (*n* = 15)). Comparing data collected using the different probe orders, count probe errors increased when count was probed last instead of first, *t*_(139)_ = 5.51, *P* < .01, potentially indicating that intervening TUT probes interfered with remembering accurate counts.  However, the replication even after interference was removed suggests the original findings were not the mere result of RT differences.

## Study 2

Regarding 1-week test-retest data, no significant practice effects were detected at the second test administration, *t*_(53)_ = .73, *P* = .47.

1. **Discussion**

## Discriminant validity: clarifying the construct of mindfulness

In moments that mindfulness is present, it may decrease stimulus-independent thought through a number of mechanisms, one of which is outlined here. Since mindfulness encourages direct perception of present experience, we suggest mindfulness may reduce task-unrelated thought as a natural byproduct of more fully saturating perceptual resources (Levinson et al., 2012; Forster and Lavie, 2009). According to Load Theory (Lavie, 2005), when a task engages the limited pool of perceptual resources, there are fewer left to automatically spill over and perceive task-irrelevant stimuli. As a result, TUT would be supplanted in early stage perceptual processing by direct perception of present experience, and thus be unable to advance to late stage processing where it might otherwise be maintained and elaborated by working memory into trains of TUT (Levinson et al., 2012; Teasdale et al., 1993; Smallwood and Schooler, 2006).

A second but not mutually exclusive interpretation of the lack of correlation between skill in mindfulness practice and working memory capacity is that of statistical suppression. Working memory may aid the initiation of mindfulness – i.e. remembering the intention to replace a wandering attention on the breath – and at the same time aid the maintenance of mind wandering about information not present (Levinson et al., 2012) at the cost of meta-awareness (Schooler et al., 2011). As a result, working memory’s opposing influences on breath counting performance may cancel.

## Criterion and incremental validity

We took steps to protect key findings from nonspecific training influences. We decreased the number of potential active ingredients in our mindfulness training: in contrast to typical mindfulness trainings that are in-person, group format, and use heterogeneous trainings that often include yoga and compassion meditation, we delivered a relatively process-pure training in breath counting individually and online.  In addition, we randomized participants to control groups including spatial n-back training, matched to breath counting in hours of practice and motivation as indexed by attrition.

Mindfulness questionnaires’ vulnerability to retrospective bias and other self-report biases may also help explain why the correlations between breath counting accuracy and the FFMQ and MAAS did not show more overlapping variance.  Indeed, modest correlations might have been expected, as they are not uncommon between self-report and behavioral measures of attentional constructs (e.g. Anderson et al., 2002).

1. **Supplementary Tables and Figures**

## Supplementary Tables

**Supplementary** **Table 1.** **Questionnaires used in Studies 1 and 2 with example items.**

| **Questionnaire** | **Sample Item** |
| --- | --- |
| Mindful Attention and Awareness Scale (MAAS) | “I rush through activities without being really attentive to them.” |
| Five Factors of Mindfulness Questionnaire (FFMQ) | “When I have distressing thoughts or images, I just notice them and let them go.” |
| Imaginal Process Inventory (IPI) | “I dream at work (or school).” |

**Supplementary** **Table 2.** **Exploratory measures in Studies 1-4.**

| **Measure** | **Statistic** |
| --- | --- |
| *Study 1* | *Counting accuracy correlation* |
| CFQ | *r* = -.22, *P* = .03 |
|  |  |
| *Study 2* | *Counting accuracy correlation* |
| ARS | *r* = -.13, *P* = .14 |
| ASRS | *r* = -.17, *P* = .05 |
|  |  |
| *Study 3* | *Counting accuracy correlation* |
| BIS | *r* = -.07, *P* = .67 |
|  |  |
| *Study 4* | *Group X Time interaction* |
| OSPAN | *F*_(2, 68)_ = 0.74, *P* = .48 |
| BDS | *F*_(2, 68)_ = 0.21, *P* = .81 |
| RMET | *F*_(2, 68)_ = 0.29, *P* = .75 |
| IPI | *F*_(2, 68)_ = 1.93, *P* = .15 |
| BIS | *F*_(2, 68)_ = 0.55, *P* = .58 |
| BDI | *F*_(2, 68)_ = 1.20, *P* = .31 |
| PWB | *F*_(2, 68)_ = 0.65, *P* = .53 |
| PANASn | *F*_(2, 68)_ = 0.69, *P* = .51 |
| PANASp | *F*_(2, 68)_ = 1.56, *P* = .22 |
| STAIX | *F*_(2, 68)_ = 1.45, *P* = .24 |
| AAQ | *F*_(2, 68)_ = 0.74, *P* = .48 |
| ARS | *F*_(2, 68)_ = 0.02, *P* = .98 |
| ASRS | *F*_(2, 68)_ = 1.97, *P* = .15 |
| PSWQ | *F*_(2, 68)_ = 1.85, *P* = .17 |
| LS | *F*_(2, 68)_ = 1.15, *P* = .32 |
| Mood ES | *F*_(2, 68)_ = 0.03, *P* = .97 |
| TUT ES | *F*_(2, 68)_ = 1.14, *P* = .33 |
| SCS | *F*_(2, 68)_ = 3.87, *P* = .03* |

*The n-back and no training control groups did not significantly differ from each other in their pre - post change in self compassion, *F*_(1, 68)_ = 2.54, *P* = .12. In contrast, the breath counting group increased in self compassion relative to the two control groups, *F*_(1, 68)_ = 5.92, *P* = .02.

CFQ = Cognitive Failure Questionnaire; ARS = Anger Rumination Scale; ASRS = Adult ADHD Self Report Scale; BIS = Barratt Impulsivity Scale; OSPAN = Operation Span; BDS = Backward Digit Span; RMET = Reading the Mind in the Eyes Task; IPI = Imaginal Process Inventory (Daydreaming subscale); BDI = Beck Depression Inventory; PWB = Psychological Wellbeing; PANASn = Positive and Negative Affect Scale, negative affect only; PANASp = Positive and Negative Affect Scale, positive affect only; STAIX = Spielberger Trait Anxiety Inventory X-2; AAQ = Acceptance and Action Questionnaire; PSWQ = Penn State Worry Questionnaire; LS = Life Satisfaction; Mood ES and TUT ES = average response to text messages 8x/day for 7 days asking “How do you feel?” on a scale of (1)=bad through (9)=good and “Are you thinking of something other than what you are doing?” in a yes/no format, respectively; SCS = Self-Compassion Scale.

**Supplementary Table 3. FFMQ subscales' correlation with breath counting accuracy.**

| **Subscale** | **Statistic*** |
| --- | --- |
| *Study 1* | *N = 93, undergraduate population* |
| Observing | *r = .03, P = .74* |
| Describing | *r = .15, P = .16* |
| Acting with awareness | *r = .21, P = .04* |
| Non-judging | *r = .11, P = .28* |
| Non-reactivity | *r = .12, P = .23* |
|  |  |
| *Study 3* | *N = 39, community sample*  *including long-term meditators* |
| Observing | *r = .13, P = .43* |
| Describing | *r = .43, P < .01* |
| Acting with awareness | *r = .26, P = .12* |
| Non-judging | *r = .34, P = .03* |
| Non-reactivity | *r = .36, P = .02* |

*Not corrected for multiple comparisons

## Supplementary Figures


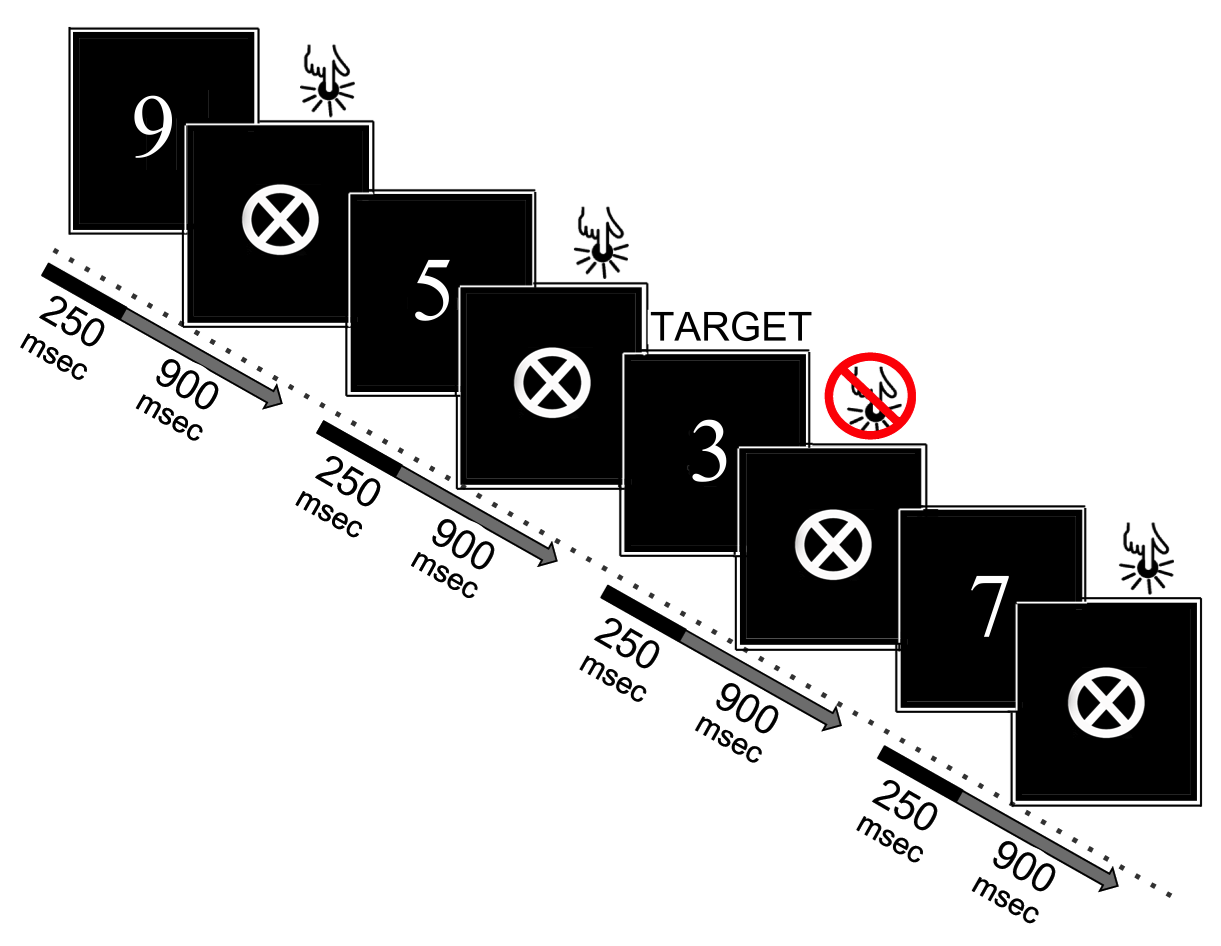


**Supplementary Figure 1.** **Sustained Attention to Response Task** schematic (adapted from Grahn and Manly, 2012).

**
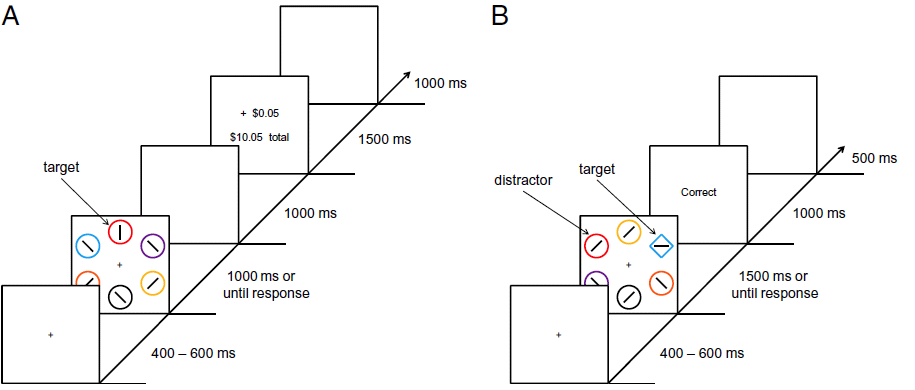
**

**Supplementary Figure 2.** **Attention capture** schematic of **(A)** training and **(B)** testing (adapted with permission from Anderson et al., 2011).

**Supplementary Figure 3.** **Diagram detailing Study 4 retention rates and reasons for dropout.**


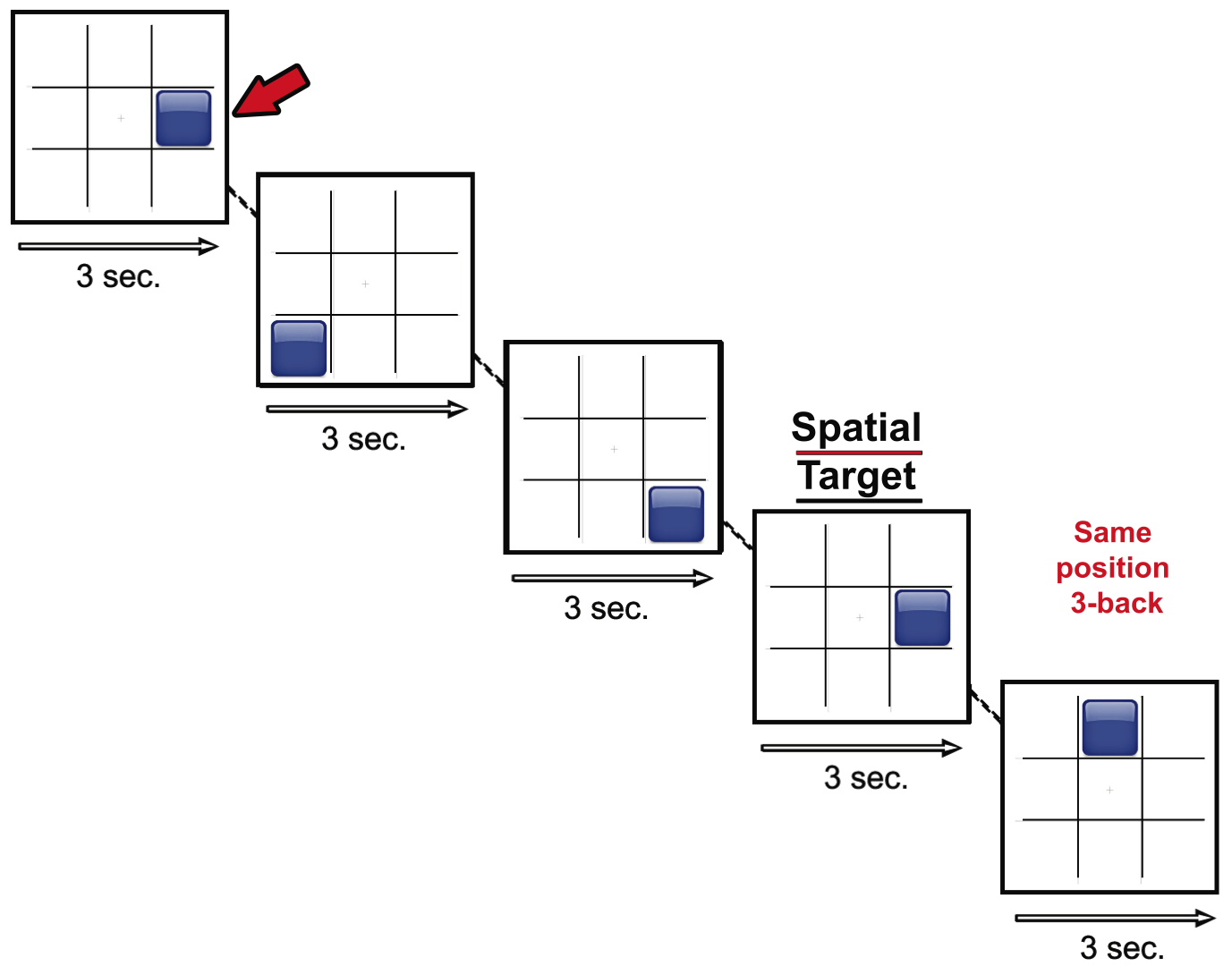


**Supplementary Figure 4.** N-back training schematic with a 3-back level exemplified.


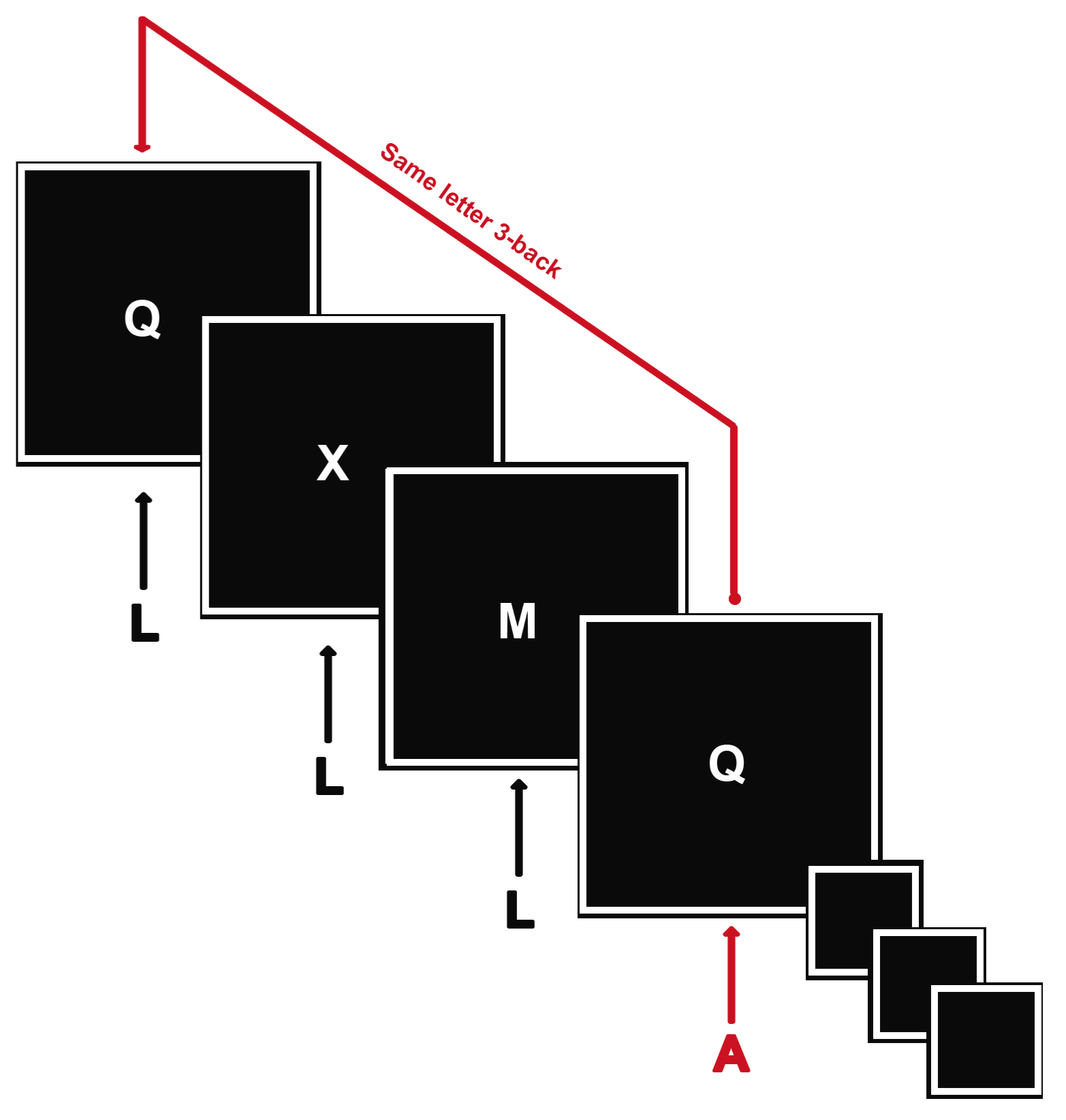


**Supplementary Figure 5.** Verbal 3-back schematic.

**Supplementary Figure 6.** Change in performance over the course of 20 consecutive weekdays of training. Performance during daily training was calculated from the average of 2 25 minute training sessions (AM and PM). For a given training session, breath counting performance was indexed as the percent of total count sets correct, and n-back performance was indexed as the highest n-back level achieved. Error bars represent within participants' +/- 1 SE.

1. **References**

Allan Cheyne, J., Solman, G. G., Carriere, J. J., and Smilek, D. (2009). Anatomy of an error: A bidirectional state model of task engagement/disengagement and attention-related errors. *Cognition* 111, 98–113. doi:10.1016/j.cognition.2008.12.009.

Anderson, B. A., Laurent, P. A., and Yantis, S. (2011). Value-driven attentional capture. *Proc. Natl. Acad. Sci. U. S. A.* 108, 10367–10371. doi:10.1073/pnas.1104047108.

Anderson, V. A., Anderson, P., Northam, E., Jacobs, R., and Mikiewicz, O. (2002). Relationships between cognitive and behavioral measures of executive function in children with brain disease. *Child Neuropsychol.* 8, 231–240. doi:10.1076/chin.8.4.231.13509.

Bodhi, B. (1993). “A Comprehensive Manual of Abhidhamma: The Abhidhammattha Sangaha of Ācariya Anuruddha,” in *A Comprehensive Manual of Abhidhamma: The Abhidhammattha Sangaha of Ācariya Anuruddha* (Kandy: BPS), 86.

Forster, S., and Lavie, N. (2009). Harnessing the wandering mind: The role of perceptual load. *Cognition* 111, 345–355. doi:10.1016/j.cognition.2009.02.006.

Grahn, J. A., and Manly, T. (2012). Common neural recruitment across diverse sustained attention tasks. *PloS One* 7, e49556. doi:10.1371/journal.pone.0049556.

Harrison, T. L., Shipstead, Z., Hicks, K. L., Hambrick, D. Z., Redick, T. S., and Engle, R. W. (2013). Working memory training may increase working memory capacity but not fluid intelligence. *Psychol. Sci.* 24, 2409–2419. doi:10.1177/0956797613492984.

Jaeggi, S. M., Buschkuehl, M., Jonides, J., and Perrig, W. J. (2008). Improving fluid intelligence with training on working memory. *Proc. Natl. Acad. Sci. U. S. A.* 105, 6829–6833. doi:10.1073/pnas.0801268105.

Jaeggi, S. M., Buschkuehl, M., Shah, P., and Jonides, J. (2014). The role of individual differences in cognitive training and transfer. *Mem. Cognit.* 42, 464–480. doi:10.3758/s13421-013-0364-z.

Lavie, N. (2005). Distracted and confused?: selective attention under load. *Trends Cogn. Sci.* 9, 75–82. doi:10.1016/j.tics.2004.12.004.

Levinson, D. B., Smallwood, J., and Davidson, R. J. (2012). The persistence of thought: Evidence for a role of working memory in the maintenance of task-unrelated thinking. *Psychol. Sci.* 23, 375–380. doi:10.1177/0956797611431465.

Robertson, I. H., Manly, T., Andrade, J., Baddeley, B. T., and Yiend, J. (1997). “Oops!”: Performance correlates of everyday attentional failures in traumatic brain injured and normal subjects. *Neuropsychologia* 35, 747–758.

Schooler, J. W., Smallwood, J., Christoff, K., Handy, T. C., Reichle, E. D., and Sayette, M. A. (2011). Meta-awareness, perceptual decoupling and the wandering mind. *Trends Cogn. Sci.* 15, 319–326. doi:10.1016/j.tics.2011.05.006.

Smallwood, J., and Schooler, J. W. (2006). The restless mind. *Psychol. Bull.* 132, 946–58. doi:10.1037/0033-2909.132.6.946.

Teasdale, J. D., Proctor, L., Lloyd, C. A., and Baddeley, A. D. (1993). Working memory and stimulus-independent thought: Effects of memory load and presentation rate. *Eur. J. Cogn. Psychol.* 5, 417. doi:10.1080/09541449308520128.

Unsworth, N., Heitz, R. P., Schrock, J. C., and Engle, R. W. (2005). An automated version of the operation span task. *Behav. Res. Methods* 37, 498–505.
